# Growth Mindset Can Reduce the Adverse Effect of Substance Use on Adolescent Reasoning

**DOI:** 10.3389/fpsyg.2019.01852

**Published:** 2019-08-13

**Authors:** Cuicui Wang, Jie Luo, Peixin Nie, Daoyang Wang

**Affiliations:** ^1^School of Educational Science, Anhui Normal University, Wuhu, China; ^2^State Key Laboratory of Cognitive Neuroscience and Learning, IDG/McGovern Institute for Brain Research, Beijing Normal University, Beijing, China; ^3^Cognitive Brain Research Unit, Faculty of Medicine, University of Helsinki, Helsinki, Finland; ^4^Cicero Learning, Faculty of Educational Sciences, University of Helsinki, Helsinki, Finland; ^5^Collaborative Innovation Center of Assessment Toward Basic Education Quality, Beijing Normal University, Beijing, China

**Keywords:** adolescents, substance use, cognitive ability, reasoning ability, growth mindset

## Abstract

The present study examined the relationship between substance use and reasoning in adolescents, and further investigated the modulation role of growth mindset on this relationship. A total of 1759 adolescents in China with substance use experience were investigated. The results showed that substance use (smoking, drinking, and illicit drug use) was negatively correlated with reasoning (*r* = −0.24 ∼−0.39, *p* < 0.01) and growth mindset (*r* = −0.18 ∼−0.32, *p* < 0.01). Regression analysis revealed that after controlling for the background variables (i.e., age, family annual income, and parents’ educational level), only illicit drug use was the significant predictor of reasoning (β = −0.325, *t* = −14.28, *p* < 0.001). The interaction effect between growth mindset and illicit drug use was also a significant predictor of reasoning (β = −0.067, *t* = −2.92, *p* = 0.004), indicating growth mindset modulated the relationship between illicit drug use and reasoning ability. Further analysis found that the negative correlation between frequency of illicit drug use and reasoning in high growth mindset group was weaker than that of low growth mindset group (*F*_(__3__,__1733__)_ = 332.51, *p* < 0.001, *f*^2^ = 0.22). This suggests that growth mindset plays a significant moderating role in the relationship between substance use and reasoning. Overall, substance use has adverse effect on adolescent reasoning, however, growth mindset could reduce this adverse effect.

## Introduction

Substance use has been a part of the young adolescent experience for several decades, and it has been identified as a public health problem that warrants serious concern (Kerwin et al., 2015). In one survey, 72% of adolescents in American reported that they had tried alcohol, 55% reported that they had been drunk, and 49% reported that they had used an illicit substance (Johnston et al., 2008). The United Nations Office on Drugs and Crime (2015) highlighted the severity of the problem and estimated that about 5% of the world’s population aged between 15 and 65 years had used drugs at least once in 1 year. In Egypt, drug use is becoming a serious problem, and nearly 6% of adolescents admit to having experimented with drugs (Philip et al., 2016). In 2012, adolescents in Nigeria were deemed to constitute the high-risk group for drug trafficking and abuse. In China, an estimated 36.1% of adolescents have tried smoking, 57.8% have tried drinking, and 6.8% have abused drugs. Thus, it is critical to assess and intervene the substance use in adolescent.

Substance use has been associated with long-term changes in cognitive function (i.e., higher-order skills responsible for selection, monitoring, and fine-tuning of goal-directed behavior) (Goldstein and Volkow, 2002; Lubman et al., 2004). Reasoning, which has been considered as one of the most important cognitive process (Fisher, 1951), is a form of thinking in which a person generalizes a general law in a specific situation or introduces a new conclusion based on existing judgments (Stanovich and West, 2000). Research has shown that substance use can lead to decreased reasoning ability (Beatty et al., 2000; Brown et al., 2000; Lyvers, 2000; Sullivan et al., 2000; Squeglia et al., 2009; Luijten et al., 2014; Shulman et al., 2018). A meta-analysis of 89 studies based on the responses of 19,930 participants found that implicit cognitive reasoning was moderately associated with substance use (Rooke et al., 2008). Another study found that substance-dependent individuals demonstrated significantly poorer fluency, working memory, reasoning, inhibition, shifting, and decision-making than healthy controls, which demonstrates the broad range of executive impairments associated with substance use (Fernandez-Serrano et al., 2010). In summary, substance use has been consistently linked to poorer cognitive ability. However, few studies examined the effect of substance use on adolescent cognitive reasoning. Indeed, adolescence marks a period of rapid development between childhood and adulthood, and involves complex social, biological, and psychological changes (Nelson et al., 2005; Blakemore, 2008). It is therefore crucial to assess the effect of substance use on adolescent cognitive reasoning.

An important concept in positive education is “mindset,” which refers to implicit beliefs about the malleability of personal attributes (Yeager and Dweck, 2012). The growth mindset is the belief that attributes such as intelligence or personality are changeable; the fixed mindset is the belief that such attributes are fixed. The growth mindset promotes resilience, and individuals with growth mindset are more likely to focus on the effort and they interpret setbacks and challenges as effective ways to improve their ability, intelligence, and experience (Dweck, 2006; Yeager and Dweck, 2012). The individuals with growth mindset have stronger motivation to try harder to cope with their challenging situations such as substance use (Elliott and Dweck, 1988). In this way, growth mindsets can moderate the link between challenging situations and subsequent performance/adjustment; in contrast, in individuals with fixed mindsets, higher difficulty corresponds with poorer performance and adjustment, whereas this association is weaker among growth-mindset individuals. The general finding that a growth mindset buffers the negative consequences of challenging and demanding environments has implications for adolescent development, such as self-regulation and goal achievement (Burnette et al., 2013; Infurna and Luthar, 2016). In addition, Han et al. (2018) found that believing growth mindset can positively influence motivation to engage in prosocial behavior, which can help individuals become a better person eventually. Considering that more impulsive and sensation-seeking adolescents are at a greater risk of early use of a variety of substances (Iacono et al., 2008). The growth mindset may be protective against the adverse effects associated with substance use in adolescents, so instilling a growth mindset is especially important for substance users who feel they lack control in their life. However, no studies to date have been conducted to examine whether and how growth mindset has a mediating effect on the adverse effects of substance use on adolescent cognition.

To further understand the effect of substance use on adolescent cognitive development, we investigated the relationship between substance use and reasoning in adolescent, which was the main goal of the present study. Further, we want to examine whether and how growth mindset has a mediating effect on the effects of substance use on adolescent’s reasoning. We hypothesized that substance use in adolescents would be correlated with cognitive reasoning, and that growth mindset moderates this relationship.

## Materials and Methods

### Participants

Participants were selected from two secondary vocational schools in Fuyang and Ma’anshan Cities of Anhui Province, China. The two schools totally included 5074 students, and divided into three grades, 40 classes in each grade. Twelve classes were selected randomly at each grade level by using cluster random sampling method. A total of 1,759 adolescents (614 female; mean age: 16.86 years; SD: 0.76) participated in the study. They reported that they have substance use experience by using the substance use questionnaire in adolescents (Siu, 2011). The distribution of the participants for each of the substance use was shown in Table 1. All participants reported that they experienced smoking or drinking, or illicit at least 1–2 times in the past month. The detailed distribution was: 793 participants reported that they experienced one kind of the substance use; 389 participants reported that they experienced two kinds of the substance use; 577 participants reported that they experienced three kinds of the substance use.

**TABLE 1:** Distribution of the participants for each substance use.

	**Smoke**	**Drink**	**Illicit drugs**
Never used	734	112	1129
Once or twice	287	895	110
Several times a month	301	447	251
Several times for every week	391	304	269
Almost everyday	46	1	0
	1759	1759	1759

No participants reported having a neurological impairment. This study was carried out in accordance with the recommendations of Institutional Review Board of Human Research Ethics Committee at Anhui Normal University. The protocol was approved by Institutional Review Board of Human Research Ethics Committee at Anhui Normal University. All subjects gave written informed consent in accordance with the Declaration of Helsinki. In addition, written informed consent was obtained from the parents of the participants under the age of 16.

### Screening Measures

The substance use questionnaire in adolescents was developed by Siu (2011). The 3-item questionnaire allows participants to report the frequency of smoking, drinking, and illicit drug (such as analgesics and stimulants) use in the past month (0 = never used; 1 = once or twice; 2 = several times a month; 3 = several times for every week; 4 = almost every day).

### Outcome Measures

#### Background Questionnaire

The background questionnaire collected demographic information such as age, gender, date of birth, *hukou* (agricultural or non-agricultural^1^), family structure (single-parent families or not), out-of-work parent, and family’s socioeconomic status. The family socioeconomic status included information about annual family income and the years of parents’ education. Family annual income was categorized as follows: 1 = < 3,000 RMB; 2 = 3,000–5,999 RMB; 3 = 6,000–9,999 RMB; 4 = 10,000–2,9999 RMB; 5 = 30,000–4,9999 RMB; 6 = 50,000–9,9999 RMB; 7 = 100,000–14,9999 RMB; 8 = 150,000–200,000 RMB; and 9 = > 200,000 RMB. The years of parents’ education level were coded as follows: 0 = did not go to school; 5 = primary school; 8 = junior high school; 11 = high school (including vocational high school, technical school, and technical secondary school); 14 = junior college; 15 = undergraduate; 18 = graduate student and PhD.

#### Substance Use Risk Profile Scale

The Substance Use Risk Profile Scale (SURPS) was developed by Woicik et al. (2009). The SURPS consists of 28 items and has four subscales, as follows: anxiety sensitivity (7 items, e.g., “I get scared when I’m too nervous”), hopelessness (8 items, e.g., “Sometimes I think I am no good at all”), sensation seeking (6 items, e.g., “I would like to skydive”), and impulsivity (7 items, e.g., “I usually act without stopping to think”). Responses were made on a 4-point Likert-type scale (completely agree/agree/disagree/completely disagree). The internal consistency coefficients of anxiety sensitivity, hopelessness, sensory seeking and impulsivity in the original English version were 0.80, 0.80, 0.70, and 0.70, respectively. With the consent of the original author (Dr. Woicik) of the scale, we translated the SURPS into Chinese, and then re-translated back into English by a fluent English speaker who is a specialist in psychology. Finally, Dr. Woicik reviewed the translated Chinese and the re-translated English versions. After finalization, the internal consistency coefficients of the Chinese version of the SURPS were 0.79, 0.72, 0.66 and 0.77 for anxiety sensitivity, hopelessness, sensory seeking and impulsivity, respectively.

#### Growth Mindset

The Growth Mindset Inventory (Dweck, 2006) was used to measure the degree of the growth mindset of responders. The Growth Mindset Inventory measures two dimensions: fixed mindset and growth mindset, totally 20 items. Responses are made on a 4-point Likert-type scale (completely disagree/disagree/agree/completely agree). A higher score on either dimension indicates a greater inclination toward the respective mindset. To calculate the total growth mindset score, the fixed mindset dimension was scored in reverse and added to the growth mindset score. A higher total growth mindset score indicates a greater inclination toward growth mindset, and a lower score is indicative of a fixed mindset (Mora, 2015). The internal consistency coefficient in the present study was 0.80.

#### Reasoning Ability Test

**FIGURE 1:**
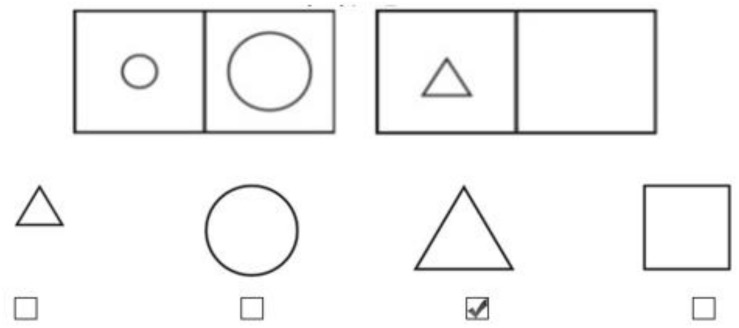
Analogical reasoning test.

**FIGURE 2:**
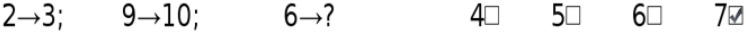
Inductive reasoning test.

The Reasoning Ability Test was developed by the National Project Team for the Investigation of Psychological Development Characteristics of Chinese Children and Adolescents (Tao et al., 2015). The Reasoning Ability Test consists of analogical reasoning (e.g., Figure 1) and inductive reasoning (e.g., Figure 2) subtests. The analogical reasoning is a composite test which consists the digital analogical reasoning and graphic analogical reasoning tasks, while the inductive reasoning only measured by the graphic sequence inference task. The internal consistency of each subtest and overall test are 0.74∼0.94.

### Quality Control

Psychology teachers or graduate students who received research training implemented the questionnaires. Four questions were asked to assess the adolescent’s surrounding environment and their psychological feelings when answering questions. These were, “Do you have any urgent thing to deal with?,” “What is your current emotional state?,” “Is there any noise or sound around you?,” and “Does the noise affect the answer?” These four questions have been shown to be a reliable basis for network test quality control (Wang and Liu, 2018). The participants who answer questions in extreme situations (such as noise interference when answering questions) will be excluded.

In addition, the substance use questionnaire relies on self-reporting, and there may be the risk of underreporting. Thus, the study also implemented the SURPS. Malmberg et al. (2010) showed that the SURPS predicts 11.4% of single substance use (such as smoking or drinking) (*R*^2^ = 0.114), while the overall illicit drug use prediction effect is 31.8% (*R*^2^ = 0.318). In the present study, the predictive effect of SURPS on smoking was 10.4% (β = 0.537, *p* < 0.01, *R*^2^ = 0.104), on drinking frequency was 17.5% (β = 0.345, *p* < 0.01, *R*^2^ = 0.175), and on the use of illicit drugs was 25.9% (β = 0.612, *p* < 0.01, *R*^2^ = 0.259), which were similar with the results of Malmberg et al. (2010). Therefore, the results of SURPS are reliable.

### Data Analysis

The data in the present study were analyzed by using SPSS20.0 and Mplus7.0. Descriptive analysis, correlation analysis, and regression analysis were included. The present study aimed to examine the effect of substance use on reasoning in adolescents, and further investigated the modulation role of growth mindset on this effect. Firstly, we did the correlation analysis to examine whether correlations were existed among the substance use, reasoning, and growth mindset. In addition, given the possible effects of background variables (such as gender, age, *hukou*, left-behind experience, family structure, and family’s socioeconomic status) on the results, we also analyzed the correlations among the background variables and substance use, reasoning, and growth mindset. Secondly, regression analysis was used to examine the independent relationships among the substance use, reasoning, and growth mindset when the background variables were controlled. Further, the interaction effect of substance use and growth mindset on reasoning was examined. Thirdly, we want to investigate how growth mindset modulated the effects of substance use on reasoning, so the simple slope test was used to examine this question.

Meanwhile, in the regression analysis, multiple collinearity tests and common-method variance tests were performed. The results of the multi-collinearity test for the variables showed that the Variance Inflation Factor (VIF) values were between 1.06 and 3.52, and there was no multicollinearity problem in the main variables according to the criterion of 0 < VIF < 10. Harman’s single factor test was used to test the common-method variance, and showed that there were four factors with an eigenvalue greater than 1; the interpretation rate of the first factor was 21.44%, which was less than the critical criterion 40%. The results indicated that the common-method variance was not significant, so it was suitable for regression analysis (Harris and Mossholder, 1996).

## Results

### Correlation Analysis

Background variables, such as gender, age, *hukou*, left-behind experience (more than half a year for both parents or one migrant worker), family structure (whether it was a single-parent family or not), and family’s socioeconomic status (family annual income and years of parents’ education), were significantly correlated with growth mindset, reasoning ability, and substance use (see Table 2). A significant positive correlation was found between growth mindset and reasoning ability (*r* = 0.34, *p* < 0.01). A significant negative correlation was found between substance use and reasoning ability (*r* = −0.24 to −0.39, *p* < 0.01), and between substance use and growth mindset (*r* = −0.18 to −0.32, *p* < 0.01).

**TABLE 2:** Demographic results and Pearson correlations between variables.

	***M***	***SD***	**1. Age**	**2**	**3**	**4**	**5**	**6**	**7**	**8**	**9**	**10**	**11**	**12**
2. Gender	0.35	0.48	–0.10^∗∗^	–										
3. *Hukou*	0.61	0.49	0.09^∗∗^	–0.10^∗∗^	–									
4. Left-behind experience	0.63	0.48	–0.04	0.02	–0.18^∗∗^	–								
5. Single-parent family	0.11	0.31	–0.04	0.01	–0.11^∗∗^	–0.14^∗∗^	–							
6. Family annual income	4.59	2.47	–0.03	–0.02	–0.11^∗∗^	0.03	0.03	–						
7. Father’s education level	9.35	3.29	−0.07^*^	0.01	–0.30^∗∗^	0.07^∗∗^	0.13^∗∗^	0.19^∗∗^	–					
8. Mother’s education level	8.82	3.57	–0.10^∗∗^	0.04	–0.35^∗∗^	0.11^∗∗^	0.13^∗∗^	0.21^∗∗^	0.66^∗∗^	–				
9. Reasoning ability	91.99	13.22	−0.07^*^	0.05^*^	0.01	–0.01	0.01	0.05	–0.08^∗∗^	–0.14^∗∗^	–			
10. Smoking frequency	1.25	1.23	0.07^∗∗^	–0.29^∗∗^	–0.02	–0.03	0.05^*^	0.11^∗∗^	0.09^∗∗^	0.11^∗∗^	–0.29^∗∗^	–		
11. Drinking frequency	1.54	0.85	0.01	–0.12^∗∗^	–0.11^∗∗^	0.02	0.04	0.16^∗∗^	0.15^∗∗^	0.19^∗∗^	–0.24^∗∗^	0.51^∗∗^	–	
12. Frequency of illicit drug use	0.81	1.17	0.06^*^	–0.19^∗∗^	–0.01	0.01	0.07^∗∗^	0.09^∗∗^	0.14^∗∗^	0.17^∗∗^	–0.39^∗∗^	0.58^∗∗^	0.64^∗∗^	–
13. Growth mindset score	32.03	3.85	–0.04	0.02	0.02	–0.05	–0.03	0.04	–0.07^∗∗^	–0.10^∗∗^	0.34^∗∗^	–0.18^∗∗^	–0.20^∗∗^	–0.32^∗∗^

Gender: male = 0, female = 1; *hukou*: non-agricultural = 0, agricultural = 1; left-behind experience and single-parent family: no = 0, yes = 1. ^*^p < 0.05, ^∗∗^*p* < 0.01, *^∗∗∗^p* < 0.001.

### Regression Analysis

The background variables such as age, family annual income, and parents’ educational level, as well as substance use and growth mindset, were used as independent variables, and reasoning ability was used as the dependent variable for the regression analysis (see Table 3).

**TABLE 3:** Regression analysis for the variables to predict reasoning without an interaction term (Model 1) and with an interaction term (Model 2).

**Variables**	**Model 1**			**Model 2**		
	***B***	***SE B***	**β**	***B***	***SE B***	**β**
Gender	0.310	0.370	0.016	0.265	0.370	0.014
Age	–0.259	0.708	–0.007	–0.332	0.707	–0.009
*Hukou*	–0.575	0.632	–0.021	–0.566	0.632	–0.036
Left-behind experience	0.220	0.600	0.008	0.240	0.600	0.009
Single-parent family	1.686	0.930	0.039	1.671	0.933	0.039
Family annual income	0.481	0.121	0.079^∗∗∗^	0.485	0.121	0.080^∗∗∗^
Father’s education level	0.143	0.107	0.031	0.151	0.107	0.033
Mother’s education level	0.101	0.105	0.022	0.100	0.105	0.022
Smoking frequency	–0.431	0.276	–0.031	–0.471	0.317	–0.034
Drinking frequency	0.028	0.316	0.002	–0.349	0.364	–0.024
Frequency of illicit drug use	–3.402	0.239	–0.325^∗∗∗^	–3.050	0.257	–0.291^∗∗∗^
Growth mindset	2.881	0.288	0.207^∗∗∗^	2.281	0.402	0.164^∗∗∗^
Smoking frequency × Growth mindset				0.230	0.505	0.013
Drinking frequency × Growth mindset				1.144	0.586	0.051
Frequency of illicit drug use × Growth mindset				–2.540	0.806	–0.067^∗∗^
*R*^2^		0.20			0.21	
*F*		60.74^∗∗∗^			46.89^∗∗∗^	

^*^p < 0.05; ^∗∗^*p* < 0.01; ^∗∗∗^*p* < 0.001.

First, in Model 1, a significant positive correlation was found between growth mindset and reasoning ability after controlling for background variables (i.e., age, family annual income, and parents’ educational level) (β = 0.207, *t* = 9.88, *p* < 0.001). Only the frequency of illicit drug use was negatively correlated with reasoning ability (β = −0.325, *t* = −14.28, *p* < 0.001). Second, in Model 2, a significant negative correlation was found between the dummy variable of the frequency of illicit drug use × growth mindset and reasoning ability (β = −0.067, *t* = −2.92, *p* = 0.004), which indicates that growth mindset modulate the relationship between the frequency of drug use and reasoning ability. In addition, the Δ*R*^2^ between Model 1 and Model 2 was significantly different (*F*_(__3__,__1733__)_ = 4.47, Δ*R*^2^ = 0.01, *p* < 0.01).

**FIGURE 3:**
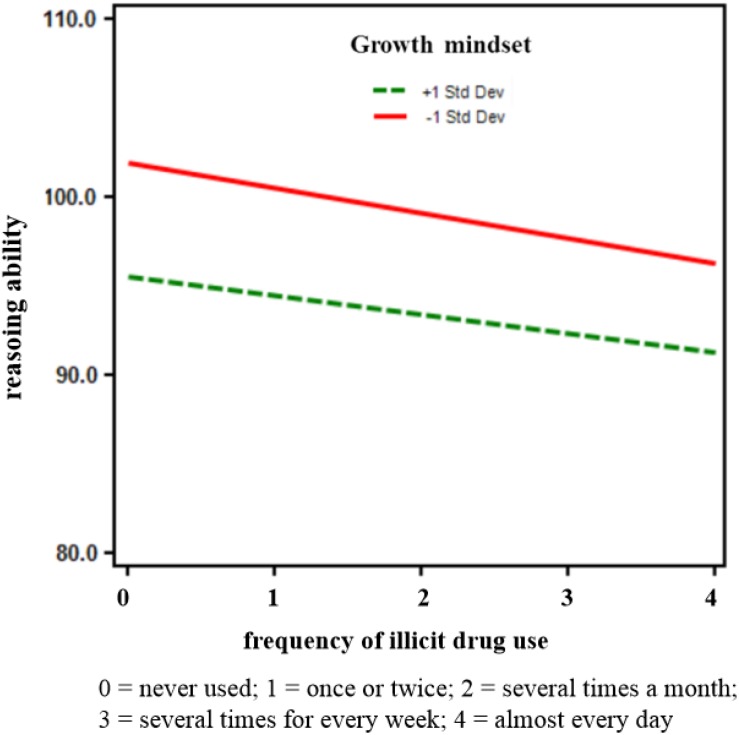
The modulation role of growth mindset in the relationship between the frequency of illicit drug use and reasoning ability in adolescents.

We further tested the modulation effect of growth mindset using the simple slope test. Growth mindset scores were divided into high and low scores according to the mean ± 1 standard deviation (Figure 3). Reasoning ability was input as the dependent variable and the frequency of illicit drug as the independent variable. Significant negative correlations were found between reasoning ability and the frequency of illicit drug use, within both the high and low growth mindset groups (high growth mindset group: simple slope = −4.23, *t* = −15.84, *p* < 0.001; low growth mindset group, simple slope = −5.70, *t* = −9.40, *p* < 0.001). However, further analysis found that the negative correlation between the frequency of illicit drugs and reasoning ability in the high growth mindset group was weaker than that of the low growth mindset group (*F*_(__3__,__1733__)_ = 332.51, *p* < 0.001, *f*^2^ = 0.22).

## Discussion

Assessing the effect of substance use on adolescent cognition ability is crucial. This study primarily investigated the relationship between substance use and reasoning ability. A negative correlation between substance use and reasoning ability was found. However, after controlling for the background variables, only illicit drug use predicted reasoning. In addition, the present study firstly found that growth mindset could modulate the relationship between the frequency of illicit drug use and reasoning. Specifically, as the growth mindset increases, the negative impact of the frequency of illicit drug use on reasoning ability will be weaken, that is, growth mindset could reduce the adverse effects of the frequency of illicit drug use on reasoning ability. Overall, substance use has adverse effect on adolescent reasoning ability, however, growth mindset could reduce this adverse effect.

The present study showed that only illicit drug use predicted reasoning after controlling for the background variables, which is consistent with previous studies. Studies have shown that the use of illicit drugs can lead to cognitive impairment, especially for working memory and visuo-spatial ability (Sullivan et al., 2000; Squeglia et al., 2009; Shulman et al., 2018). Thus, substance use did have an adverse effect on the adolescent cognitive development. However, unlike previous studies (Steinberg, 2005), we did not find smoking/drinking have a predictive effect on reasoning. The reason may be that the adolescents only have a short history of smoking or drinking, which may not have been sufficient to detect degrees of damage to long term reasoning ability.

For the first time, we found that growth mindset modulated the relationship between illicit drug use and reasoning ability. The growth mindset believes that traits are malleable and the potential for change is possible (Yeager and Dweck, 2012). People with growth mindset tend to work harder and exert more effort to reduce the substance use, which diminished the negative impact of substance use on reasoning ability. Previous study found that growth mindset even could reduce the desire for revenge after hypothetical peer victimization (Yeager et al., 2011). Thus, educators and parents may consider implementing interventions targeting growth mindset in order to reduce challenging behavioral by making students believe that their behavioral can be changed by effort. For adolescents who have substance use behavioral, if their teacher or parents constantly instill growth mindset for them, then they will think that their behavior can be changed through efforts. So, on one hand, they may reduce substance use, and on the other hand, they may seriously invest in learning and improve their cognitive development. Therefore, strengthening adolescents’ awareness that attributes are changeable can decrease the negative impact of the frequency of illicit drug use on reasoning ability. However, despite this significant moderating effect of growth mindset, the specific effect value was still small (Δ*R*^2^ = 0.01), accounting for only 5% of the effect (0.01/0.20 = 0.05). The reason may be that adolescents are at a stage that their trait has not yet stabilized, so their growth mindset may not be stable.

Overall, these results may have important educational implications for adolescents. Schools, society, and families should pay attention to the restrictions of substance use in adolescents. Adolescents who use substances can be instilled with the concept of growth mindset by changing their conscious attitudes, expectancies, and beliefs to minimize the negative effects of substance use on reasoning ability. However, some limitations and suggestions should be noted in the present study. First, the present study only investigated the students from secondary vocational schools. However, substance use may be more prevalent in secondary vocational schools compared with academic high schools (Ling, 2015). The academic performance of students in secondary vocational schools is much lower than those in academic high schools. In addition, the students in vocational schools more socialized and it is common for smoking and drinking behavioral in vocational school (Ling, 2015). Thus, further research could expand the types of schools to be sampled and conduct surveys in academic high schools. Second, different types of substances may have different effects on reasoning ability, and this could be investigated in subsequent studies. Finally, considering the negative impact of substance use on reasoning ability is the result of long-term accumulation, so the duration of substance use should not be ignored. Future longitudinal studies could examine the stability of the results in the present study.

## Data Availability

The datasets for this manuscript are not publicly available because the present study was funded by the agencies that did not authorize the disclosure of normative data. Requests to access the datasets should be directed to the National Social Science Fund of China, Email: daoyang@ahnu.edu.cn.

## Ethics Statement

This study was carried out in accordance with the recommendations of Institutional Review Board of Human Research Ethics Committee at Anhui Normal University with written informed consent from all subjects. All subjects gave written informed consent in accordance with the Declaration of Helsinki. The protocol was approved by Institutional Review Board of Human Research Ethics Committee at Anhui Normal University.

## Author Contributions

CW: study design, literature research, data acquisition, data analysis, and manuscript writing. JL: data acquisition, data analysis, and manuscript preparation. PN: literature research, manuscript editing, and manuscript revision. DW: literature research, guarantor of integrity of entire study, study design, manuscript definition of intellectual content, manuscript editing, and manuscript revision.

## Conflict of Interest Statement

The authors declare that the research was conducted in the absence of any commercial or financial relationships that could be construed as a potential conflict of interest.

## Footnotes

1 The *hukou* registration system was implemented in the 1950s in mainland China. The two types of *hukou*, namely, urban and rural, pertain to urban and rural population, respectively. *Hukou* can also be classified into non-agricultural and agricultural *hukou* based on individual’s entitlement to state-subsidized food grain. Although agriculture *hukou* exists in urban areas and non-agricultural *hukou* exists in rural areas, these cover only a negligible share of the population in these areas. Hence, we ignore this distinction and equate urban *hukou* with non-agricultural *hukou* and rural *hukou* with agriculture *hukou* (Liu, 2005).
